# Characteristics and Outcomes of Coronavirus Disease Patients under Nonsurge Conditions, Northern California, USA, March–April 2020

**DOI:** 10.3201/eid2608.201776

**Published:** 2020-08

**Authors:** Jessica Ferguson, Joelle I. Rosser, Orlando Quintero, Jake Scott, Aruna Subramanian, Mohammad Gumma, Angela Rogers, Shanthi Kappagoda

**Affiliations:** Stanford Health Care, Stanford, California, USA (J. Ferguson, J.I. Rosser, O. Quintero, J. Scott, A. Subramanian, A. Rogers, S. Kappagoda);; Stanford University, Stanford (M. Gumma)

**Keywords:** Coronavirus diseases, coronavirus disease, COVID-19, SARS-CoV-2, severe acute respiratory syndrome coronavirus 2, respiratory diseases, zoonoses, viruses, pneumonia, hospitalization, ICU, mortality, outcomes, California, United States

## Abstract

Limited data are available on the clinical presentation and outcomes of coronavirus disease (COVID-19) patients in the United States hospitalized under normal-caseload or nonsurge conditions. We retrospectively studied 72 consecutive adult patients hospitalized with COVID-19 in 2 hospitals in the San Francisco Bay area, California, USA, during March 13–April 11, 2020. The death rate for all hospitalized COVID-19 patients was 8.3%, and median length of hospitalization was 7.5 days. Of the 21 (29% of total) intensive care unit patients, 3 (14.3% died); median length of intensive care unit stay was 12 days. Of the 72 patients, 43 (59.7%) had underlying cardiovascular disease and 19 (26.4%) had underlying pulmonary disease. In this study, death rates were lower than those reported from regions of the United States experiencing a high volume of COVID-19 patients.

Health officials in China first reported a cluster of cases of a new acute respiratory illness associated with a seafood market in Wuhan, China, on December 31, 2019 (*1*). Less than 1 month later, cases of what would become known as coronavirus disease (COVID-19) were reported in patients in northern California, USA (*2*). In the San Francisco Bay area counties of Alameda and Santa Clara, COVID-19 cases in travelers returning from Wuhan were confirmed on January 28 and January 31, respectively. As of May 5, 2020, a total of 1,809 laboratory-confirmed cases have occurred in Alameda County (population 1.7 million) and 2,555 cases in Santa Clara County (population 1.9 million); however, these numbers probably vastly underestimate the disease incidence because of the lack of widespread testing in the region early in the epidemic (*3*,*4*). Studies from China and Europe have described the clinical presentation of COVID-19, but data from the United States are still emerging (*5**–**9*). In addition, current data from the United States have primarily come from hospitals working under high-volume or surge conditions. In this study, we describe the characteristics and outcomes of patients hospitalized in northern California with COVID-19 early in the epidemic under nonsurge conditions.

## Methods

### Study Design and Oversight

We conducted a retrospective chart review of demographic and clinical data for patients admitted to our 2 partner institutions, Stanford University Hospital (SUH) and Stanford Health Care-ValleyCare (ValleyCare), during March 13–April 11, 2020, with follow up through May 2, 2020. SUH is an academic medical center with 600 beds, including 119 intensive care unit (ICU) beds, located in Palo Alto, Santa Clara County. ValleyCare is a community hospital with 167 beds, including 22 ICU beds, in Pleasanton, Alameda County. The Stanford Health Care Institutional Review Board approved this study.

### Inclusion and Exclusion Criteria

We sequentially enrolled all patients >18 years of age who were hospitalized for >24 hours and had reverse transcription PCR (RT-PCR)–confirmed severe acute respiratory syndrome coronavirus 2 (SARS-CoV-2) during the study period. Our study comprised patients who spent >1 nights in the hospital; we excluded patients seen only in the emergency department and discharged in <24 hours.

### Data Sources and Collection

We used the electronic medical record system, EpicSystems (https://www.epic.com) to extract data on clinical symptoms and signs, laboratory test results, and outcomes. We collected and managed study data using REDCap electronic data capture tools hosted at Stanford University (*10*,*11*).

### Laboratory Testing

All laboratory data were ordered as part of routine clinical care. SARS-CoV-2 infection was confirmed using RT-PCR, which was validated on nasopharyngeal, oropharyngeal, endotracheal aspirate, and bronchoalveolar lavage samples.

### Definitions

We recorded the date of the earliest reported symptom. Diabetes was defined by any preadmission medication prescription for diabetes or documented hemoglobin A1C >6.5% (reference <5.7%). Admission tests refers to laboratory studies collected within the first 24 hours of admission. We defined admission chest radiograph as one done within 24 hours after hospital arrival and admission chest computed tomography as one performed within 48 hours after hospital arrival. Radiographic findings were based on the radiology report in the electronic medical record. We defined acute respiratory distress syndrome using the Berlin Criteria (presence of acute respiratory failure with bilateral pulmonary infiltrates, ratio of arterial oxygen tension to fraction of inspired oxygen <300 with >5 cm water of positive-end expiratory pressure, and absence of cardiogenic pulmonary edema) (*12*). We defined acute kidney injury as an increase in serum creatinine during admission of 1.5 times baseline (*13*). We defined cardiomyopathy as an ejection fraction assessed on transthoracic echocardiogram of <50% or >10% decrease from the baseline ejection fraction if the result of a prior echocardiogram within the past 2 years was available. We defined central line–associated bloodstream infection using National Healthcare Safety Network criteria (*14*).

### Data Analysis

We conducted all analyses using R version 3.6.2 (https://www.r-project.org). The 4 patients who remained hospitalized at the end of the study period were right-censored on May 2 and included in length-of-stay calculations. We performed Mann-Whitney U tests for continuous variables and Fisher exact tests for categorical variables, all using a type I error of 0.05.

## Results

### Demographic Characteristics

A total of 72 SARS-CoV-2–positive patients were admitted during March 13–April 11, 2020. Twelve were admitted to ValleyCare Hospital and 60 to Stanford Hospital. Twenty-two (30.6%) patients were Hispanic or Latino, 20 (27.8%) were Asian or Asian-American, 19 (26.4%) were white, and 4 (5.6%) were black (Table 1). Most (51 [70.8%]) patients came from stable housing situations with family. Twelve (16.7%) lived in a skilled nursing facility, assisted living, group home, or unstable living situation, and 1 (1.4%) lived alone.

**Table 1:** Baseline demographic and clinical characteristics of persons with COVID-19, northern California, 2020*

Characteristic	COVID-19 patients			OR (95% CI) or p value
	All, n = 72	Non-ICU, n = 51	ICU, n = 21	
Median age, y (IQR)	60.4 (43.4–70.6)	61.7 (46.6–72.9)	57.6 (42.2–70.1)	0.39
Sex, no. (%)				
M	38 (52.8)	25 (49.0)	13 (61.9)	1.68 (0.54–5.54)
F	34 (47.2)	26 (51.0)	8 (38.1)	
Race/ethnicity, no. (%)				
Hispanic/Latino	22 (30.6)	13 (25.5)	9 (42.9)	2.54 (0.54–14.07)
Asian	20 (27.8)	13 (25.5)	7 (33.3)	0.58 (0.12–2.17)
Black	4 (5.6)	3 (5.9)	1 (4.8)	1.24 (0.02–21.44)
White, non-Hispanic	19 (26.4)	15 (29.4)	4 (19.0)	Referent
Other	2 (2.8)	2 (3.9)	0	NP
Unknown	5 (6.9)	5 (9.8)	0	NP
Smoking history, no. (%)				
Ever	17 (27.4)	11 (25.6)	6 (31.6)	1.34 (0.33–5.0)
Never	45 (72.6)	32 (74.4)	13 (68.4)	
Housing, no. (%)				
Stable, with others in the home	51 (70.8)	37 (72.5)	14 (66.7)	0.85 (0.22–3.21)
SN/LTACH facility	8 (11.1)	6 (11.8)	2 (9.5)	
Group home	3 (4.2)	3 (5.8)	0	
Stable, living alone	1 (1.4)	0	1 (4.8)	
Unstable	1 (1.4)	0	1 (4.8)	
Unknown	8 (11.1)	5 (9.8)	3 (14.3)	
Sick contacts, no. (%)				
Confirmed COVID-19	25 (34.7)	16 (31.4)	9 (42.9)	2.08 (0.72–6.00)†
PUI contact	14 (19.4)	9 (17.6)	5 (23.8)	
None	33 (45.8)	26 (51.0)	7 (33.3)	
Preexisting concurrent conditions, no. (%)				
Obesity				
BMI >30	24 (36.4)	14 (30.4)	10 (50.0)	2.26 (0.68–7.67)
BMI <30	42 (63.6)	32 (69.6)	10 (50.0)	
Cardiovascular disease				
Any	43 (59.7)	28 (54.9)	15 (71.4)	2.03 (0.62–7.47)
Hypertension	26 (36.1)	15 (29.4)	11 (52.4)	2.60 (0.81–8.54)
Hyperlipidemia	25 (34.7)	17 (33.3)	8 (38.1)	1.23 (0.36–3.96)
Diabetes	20 (27.8)	10 (19.6)	10 (47.6)	3.65 (1.07–12.84)
Chronic kidney disease‡	9 (12.5)	7 (13.7)	2 (9.5)	0.67 (0.06–3.95)
Coronary artery disease§	7 (9.7)	5 (9.8)	2 (9.5)	0.97 (0.09–6.58)
Arrhythmia	6 (8.3)	3 (5.9)	3 (14.3)	2.63 (0.32–21.55)
Heart failure	5 (6.9)	4 (7.8)	1 (4.8)	0.59 (0.01–6.48)
Cerebrovascular disease	2 (2.8)	1 (2.0)	1 (4.8)	2.46 (0.03–199.75)
Other	6 (8.3)	6 (11.8)	0	NP
Pulmonary disease				
Any	19 (26.4)	15 (29.4)	4 (19.0)	0.56 (0.16–1.96)
COPD/asthma	10 (13.9)	7 (13.7)	3 (14.3)	
Other¶	10 (13.9)	9 (17.6)	1 (4.8)	
Immunocompromise#	6 (8.3)	5 (9.8)	1 (4.8)	0.46 (0.01–4.55)
Preadmission medications, no. (%)				
Cardiovascular medication				
Any	30 (41.7)	20 (39.2)	10 (47.6)	1.40 (0.44–4.42)
Statin	17 (23.6)	10 (19.6)	7 (33.3)	2.03 (0.54–7.32)
CCB	13 (18.1)	8 (15.7)	5 (23.8)	1.67 (0.37–6.85)
Beta-blocker	9 (12.5)	6 (11.8)	3 (14.3)	1.25 (0.18–6.63)
Diuretic	9 (12.5)	7 (13.7)	2 (9.5)	0.67 (0.06–3.95
ARB	8 (11.1)	4 (7.8)	4 (19.0)	2.72 (0.45–16.39)
ACE-1	5 (6.9)	2 (3.9)	3 (14.3)	3.99 (0.42–51.42)
Digoxin	1 (1.4)	1 (2.0)	0	NP
Other	3 (4.2)	1 (2.0)	2 (9.5)	NP
Inhaled respiratory medication	21 (29.2)	13 (25.5)	8 (38.1)	1.78 (0.52–5.97)
Steroid inhaler	13 (18.1)	8 (15.7)	5 (23.8)	
Tiotropium	9 (12.5)	6 (11.8)	3 (14.3)	
LABA	8 (11.1)	4 (7.8)	4 (19.0)	
SABA	5 (6.9)	2 (3.9)	3 (14.3)	
Immunosuppressant				
Oral glucocorticoids	1 (1.4)	1 (2.0)	0	NP
Other**	6 (8.3)	5 (9.8)	1 (4.8)	0.46 (0.01–4.55)
Azithromycin	20 (27.8)	14 (27.5)	6 (28.6)	0.95 (0.27– 3.59)
Hydroxychloroquine	2 (2.8)	2 (3.9)	0	NP

*ACE-I, angiotensin-converting-enzyme inhibitor; ARB, angiotensin-receptor blocker; BMI, body mass index; COPD, chronic obstructive pulmonary disease; CCB, calcium channel blocker; COVID-19, coronavirus disease; ICU, intensive care unit; IQR, interquartile range; LABA, long-acting inhaled β-agonist, LTAC, long-term acute care; NP, test not performed; PUI, person under investigation; SABA, short-acting inhaled β-agonist; SNF, skilled nursing. †Compares confirmed COVID-19 contact or PUI contact with no sick contact. ‡Comprises 1 patient on outpatient hemodialysis and 8 patients not on hemodialysis. §Any history of coronary artery bypass surgery, coronary artery stenting, or clinical diagnosis of coronary artery disease according to the electronic medical record. ¶Cystic fibrosis, interstitial lung disease, pulmonary hypertension, and obstructive sleep apnea. #Active malignancy, history of solid organ transplantation, or autoimmune disease. **Mammalian target of rapamycin inhibitors, calcineurin inhibitors, imatinib, lenalidomide, regorafenib, and paclitaxel.

### Concurrent Conditions

Forty-three (59.7%) patients had underlying cardiovascular disease, and 19 (26.4%) had underlying pulmonary disease. Thirty-six percent had no cardiovascular or pulmonary disease. Among the ICU patients, in univariate analysis, only diabetes was significantly associated with ICU admission.

The most common concurrent conditions among all 72 patients were hypertension (36.1%), hyperlipidemia (34.7%), and diabetes (27.8%). Twenty-three non-ICU (45.1%) and 6 ICU (28.6%) patients had no known cardiovascular disease. The most common respiratory concurrent conditions were asthma (7 patients) or chronic obstructive pulmonary disease (3). Tobacco use did not differ between non-ICU and ICU patients.

Six (8.3%) patients had an immunocompromising condition. These conditions included 3 with active malignancies, 2 solid organ transplant recipients, and 1 patient with systemic sclerosis.

Before admission, 20 (27.8%) patients had been prescribed azithromycin. Two (2.8%) patients had been prescribed hydroxychloroquine to target COVID-19. No patients were on long-term hydroxychloroquine for other indications. Twenty-six (36.1%) patients, including 5 (23.8%) treated in the ICU, had no known concurrent conditions.

### Characteristics at Admission

At admission, the most common symptoms were fever (73.6%), dry cough (58.3%), and shortness of breath (56.9%) (Table 2). Patients also commonly reported nonspecific influenza-like symptoms of fatigue, myalgias, nausea, and diarrhea. Few (5.6%) patients reported altered sensation of taste or smell. ICU patients were more likely to have fever documented within 24 hours after admission and had higher temperatures recorded. Of 48 patients who had a multiplex PCR–based respiratory viral panel performed within 24 hours after admission, 2 had a viral co-infection (1 patient with respiratory syncytial virus and 1 with both respiratory syncytial virus and rhinovirus). 

**Table 2:** Clinical characteristics and laboratory and radiographic findings for COVID-19 patients, northern California, 2020

Characteristic	COVID-19 patients			p value
	All, n = 72	Non-ICU, n = 51	ICU, n = 21	
Symptoms, no. (%)				
Fever	53 (73.6)	37 (72.5)	16 (76.2)	1.00
Chills	25 (34.7)	15 (29.4)	10 (47.6)	0.18
Cough, dry	42 (58.3)	28 (54.9)	14 (66.7)	0.44
Cough, productive	15 (20.8)	12 (23.5)	3 (14.3)	0.53
Shortness of breath	41 (56.9)	29 (56.9)	12 (57.1)	1.00
Chest pain/pressure	8 (11.1)	6 (11.8)	2 (9.5)	1.00
Fatigue	26 (36.1)	21 (41.2)	5 (23.8)	0.19
Myalgias	32 (44.4)	19 (37.3)	13 (61.9)	0.07
Arthralgias	1 (1.4)	1 (2.0)	0	1.00
Headache	14 (19.4)	9 (17.6)	5 (23.8)	0.53
Sore throat	10 (13.9)	5 (9.8)	5 (23.8)	0.14
Nasal congestion/rhinorrhea	7 (9.7)	3 (5.9)	4 (19.0)	0.18
Nausea	17 (23.6)	15 (29.4)	2 (9.5)	0.13
Vomiting	7 (9.7)	7 (13.7)	0	0.01
Diarrhea	19 (26.4)	13 (25.5)	6 (28.6)	0.78
Altered sense of taste/smell	4 (5.6)	3 (5.9)	1 (4.8)	1.00
Other†	16 (22.2)	11 (21.6)	5 (23.8)	NP
Vital signs				
Medium max temp in first 24 h, °C (IQR)	38.1 (37.3–38.8)	37.8 (37.2–38.7)	38.6 (38.6–39.3)	<0.05
Temperature >38.2°C, no. (%)	34 (47.2)	19 (37.3)	15 (71.4)	<0.05
Room air SaO2, no. (%)				
SaO2 >94	42 (58.3)	36 (70.6)	6 (28.6)	<0.05
Median SaO2	30 (41.7)	15 (29.4)	15 (71.4)	
<94 RR (IQR)	20 (18–22)	19 (18–20)	22 (18–27)	<0.05
Laboratory results, median (IQR)‡				
Leukocytes, K/μL	5.6 (4.3–7.8)	5.7 (4.4–8.1)	5.2 (4.0–7.0)	0.40
ANC, n = 71	3,890 (2,705–5,835)	3,875 (2,630–5,725)	4140 (2930–6430)	0.42
ALC, n = 71	910 (580–1,235)	915 (592–1,335)	890 (520–1,090)	0.47
Platelets, K/μL	194 (160–256)	198 (162–265)	183 (157–250)	0.40
Sodium, mmol/L	136 (133–138)	136 (132–139)	136 (134–137)	1.00
Potassium, mmol/L	3.8 (3.7–4.2)	3.9 (3.7–4.2)	3.8 (3.6–4.0)	0.31
Creatinine, mg/dL	0.89 (0.67–1.07)	0.89 (0.73–1.07)	0.89 (0.66–1.07)	0.69
Glucose, mg/dL	108 (98–124)	107 (96–120)	114 (102–147)	0.18
AST, U/L	45.5 (31.8–63.5)	45.0 (29.0–59.5)	52.0 (38.0–82.0)	0.04
ALT, U/L	36.5 (23.8–56.2)	35.0 (22.5–51.5)	49.0 (34.0–58.0)	0.09
CK, total, U/L	119 (55–360)	53 (48–70)	282 (174–774)	0.01
LDH, U/L	394 (251–492)	344 (250–442)	430 (299–522)	0.03
Ferritin, ng/mL	824 (453–1643)	612 (304–1030)	1422 (817–1944)	0.04
CRP >0.5 ng/dL, no./total (%)	36/41 (87.8)	21/26 (80.8)	15/15 (100)	0.14
IL-6 >5 pg/mL, no./total (%)	5/7 (71.4)	3/3 (100)	2/4 (50.0)	0.43
Procalcitonin >0.5 ng/mL, no./total (%)	4/47 (8.5)	3/33 (9.1)	1/14 (7.1)	1.0
D-dimer >0.5 μg/mL, no./total (%)	20/26 (76.9)	14/19 (73.7)	6/7 (85.7)	1.0
Troponin >0.055 ng/mL, no./total (%)	2/45 (4.4)	1/31 (3.2)	1/14 (7.1)	0.53
Radiology, no. (%)				
Chest radiograph				
Diffuse/patchy bilateral infiltrates	45 (62.5)	26 (51.0)	19 (90.5)	<0.05§
Focal consolidation	11 (15.3)	9 (17.6)	2 (9.5)	NP
Pleural effusion	4 (5.6)	2 (3.9)	2 (9.5)	
Clear	11 (15.3)	11 (21.6)	0	
Other¶	5 (6.9)	4 (7.8)	1 (4.8)	
Chest computed tomography scan				
Diffuse/multifocal/GGO/opacities	11 (15.3)	8 (15.7)	3 (14.3)	
Diffuse consolidations	4 (5.6)	3 (5.9)	1 (4.8)	
Focal consolidation	2 (2.8)	2 (3.0)	0	

*ALC, absolute lymphocyte count; ALT, alanine aminotransferase; ANC, absolute neutrophil count; AST, aspartate aminotransferase; CK, creatinine kinase; COVID-19, coronavirus disease; CRP, C-reactive protein; GGO, ground glass opacities; IL-6, interleukin-6; IQR, interquartile range; LDH, lactate dehydrogenase; NP, test not performed; RR, respiratory rate. †Altered mental status, dizziness, night sweats, anorexia, and abdominal pain. ‡Reference ranges: leukocytes, 4.0–11.0 K/μL; ANC, 1,700–6,700 cells/μL; ALC, 1,000–3,000 cells/μL; platelets, 150–400 K/μL; sodium, 135–145 mmol/L; potassium, 3.5–5.5 mmol/L; creatinine, 0.67–1.17 mg/dL; glucose, 70–100 mg/dL; AST, 10–50 U/L; ALT, 10–50 U/L; CK, total <190 U/L; LDH, 135–225 U/L; ferritin 30–400 ng/mL; CRP <0.5 ng/dL; IL-6, <5 pg/mL; procalcitonin, <0.5 ng/mL; D-dimer, <0.5 μg/mL; troponin <0.055 ng/mL. §Compares diffuse/patchy bilateral infiltrates with all other categories combined. ¶Bibasilar opacities and interstitial markings.

Abnormal results of chest imaging were more common in patients requiring ICU admission. Among patients admitted to the ICU, 19 (90.5%) had an initial chest radiograph with diffuse or patchy bilateral opacities, compared with 26 (51.0%) of non-ICU patients. None of the patients admitted to the ICU had normal-appearing chest radiograph result at admission. Overall, very few patients had a chest computed tomography scan performed within the first 48 hours of hospitalization.

### Treatments

Patients most commonly received remdesivir (44.4% of all patients and 76.2% of ICU patients), azithromycin, or both during hospitalization (Table 3). Four ICU patients received tocilizumab, and 1 received leronlimab. Among the 12 patients seen at ValleyCare, in addition to supportive care, 11 (91.7%) received hydroxychloroquine, 11 (91.7%) received zinc, and 5 (41.7%) received azithromycin. No patients received convalescent plasma, lopinavir/ritonavir, chloroquine, or intravenous gamma globulin during the study period. A trial of prone positioning was used for 2 hypoxic, nonintubated patients, in accordance with institutional protocol (*15*).

**Table 3:** Complications, interventions, and outcomes of COVID-19 patients, northern California, 2020*

Variable	COVID-19 patients			p value
	All, n = 72	Non-ICU, n = 51	ICU, n = 21	
Complications, no. (%)				
Acute respiratory distress syndrome	13 (18.0)	0	13 (61.9)	NP
Arrhythmia†	6 (8.3)	0	6 (28.6)	
Ventilator- or hospital-associated pneumonia	5 (6.9)	0	5 (23.8)	
Acute kidney injury	4 (5.6)	0	4 (19.0)	
Catheter-related bloodstream infection	2 (2.8)	0	2 (9.5)	
Cardiomyopathy‡	2 (2.8)	0	2 (9.5)	
Highest level of oxygen support required, no. (%)				
None	29 (40.3)	28 (54.9)	1 (4.8)	Referent
Oxygen by nasal cannula	22 (30.6)	21 (41.2)	1 (4.8)	1.0
High-flow nasal cannula	2 (2.8)	1 (2.0)	1 (4.8)	0.13
Nonrebreather mask	6 (8.3)	1 (2.0)	5 (23.8)	<0.05
Mechanical ventilation	13 (18.1)	0	13 (61.9)	NP
Median duration of mechanical ventilation, d§ (IQR)		NP	17 (13–29)	NP
Interventions, no. (%)				
Use of a paralytic agent	7 (9.7)	0	7 (33.3)	NP
Use of proning	6 (8.3)	1 (2.0)	5 (23.8)	
Tracheostomy	6 (8.3)	0	6 (28.6)	
Use of vasopressors	13 (18.1)	0	13 (61.9)	
Use of renal replacement therapy¶	4 (5.6)	0	4 (19.0)	
Inhaled nitric oxide	4 (5.6)	0	4 (19.0)	
Treatment, no. (%)				
Azithromycin	33 (45.8)	19 (37.3)	14 (66.7)	<0.05
Remdesivir	32 (44.4)	15 (29.4)	16 (76.2)	<0.05
Hydroxychloroquine	16 (22.2)	11 (21.6)	5 (23.8)	1.0
Systemic glucocorticoids	5 (6.9)	3 (5.9)	2 (9.5)	0.63
Tocilizumab	4 (5.6)	0	4 (19.0)	<0.05
Other#	11 (15.3)	8 (15.7)	3 (14.3)	NP
Any antimicrobial drug	48 (66.7)	28 (54.9)	19 (90.5)	<0.05
Any antifungal drug	1 (1.4)	0	1 (4.8)	0.29
Median length of stay,** d (IQR)				
Hospitalization	7.5 (4–13)	5 (3–9)	17 (11–30)	
ICU	NP	NP	12 (5–28)	
Disposition, no. (%)				
Discharged from hospital				
Home	53 (73.6)	43 (84.3)	10 (47.6)	0.35††
SN/LTAC facility	9 (12.5)	5 (9.8)	4 (19.0)	
Died or discharged with hospice	6 (8.3)	3 (5.9)	3 (14.3)	
Remains hospitalized	4 (5.6)	0	4 (19.0)	

*COVID-19, coronavirus disease; ICU, intensive care unit; IQR, interquartile range; LTAC, long-term acute care; NP, test not performed; SN, skilled nursing. †Includes 2 atrial fibrillation with rapid ventricular response, 2 supraventricular tachycardia, 2 bradycardia. ‡New ejection fraction <50% after previously normal ejection fraction on echocardiogram in the preceding 2 y and/or >10% decrease in ejection fraction from baseline. §Includes 1 patient who remained on mechanical ventilation at discharge to LTAC facility on April 7, 2020. ¶Newly requiring renal replacement therapy during admission. #Zinc, n = 11; leronlimab, n = 1. **Length of stay includes 4 patients who remained hospitalized on the study end date with censoring date of May 2, 2020. ††Comparison of death or discharged with hospice in ICU and non-ICU patients (Fisher exact test).

### Complications

No major complications developed in the patients who never required ICU care during hospitalization (non-ICU patients) (Table 3). Acute respiratory distress syndrome developed in 13 (61.9%) ICU patients. No patients were treated with intravenous pulmonary vasodilators or extracorporeal membrane oxygenation. Arrhythmias developed in 6 ICU patients, 4 with supraventricular tachycardia, including atrial fibrillation or atrial flutter, and 2 with bradycardia, including complete heart block requiring a permanent pacemaker that developed in 1.

### Outcomes

Five (6.9%) patients died, and 1 patient was discharged to hospice. Fifty-three (73.6%) patients were discharged home, and 9 (12.5%) were discharged to a skilled nursing facility or long-term acute care hospital. At the end of the study period (May 2, 2020), 4 remained hospitalized awaiting placement in a skilled nursing facility or long-term acute care hospital. Of the 6 patients who died or were discharged to hospice, median age was 83.5 years. Median length of hospitalization for all patients was 7.5 days (interquartile range [IQR] 4–13 days).

Among ICU patients, 3 (14.3%) died; median length of hospitalization was 17 days (IQR 11–30 days). Thirteen (61.9%) ICU patients required mechanical ventilation for a median of 17 days (IQR 13–29 days), and 6 (28.6%) patients underwent tracheostomy. All 4 patients who remained hospitalized had required ICU admission; however, all had improved by the end of the study period, and none still required mechanical ventilation or ICU level of care.

## Discussion

We found a lower overall death rate (8.3%) than for the largest US studies thus far, which reported 17.5%–21% death rates (J.A. Lewnard et al., unpub. data, https://doi.org/10.1101/2020.04.12.20062943; C.M. Petrilli et al., unpub. data, https://doi.org/10.1101/2020.04.08.20057794) (*16*). The death rate for ICU patients of 14.3% was also lower than previously reported rates within the United States (45%–50%) (C.M. Petrilli et al., unpub. data, https://doi.org/10.1101/2020.04.08.20057794) (*17*). These results are based on censoring 4 patients who remained hospitalized at the end of the study period, all of whom no longer required ICU level of care and awaited placement in rehabilitation or long-term acute care facilities. Although our sample size is small, this difference in death rates might be attributed to nonsurge conditions. Patient volumes did not exceed normal operating capacity in the 2 hospitals during the study period, which might signify that patients were admitted who might not merit hospitalization in conditions where hospital beds were limited. Disease severity was probably lower in the population in our study; 71% (51/72) of patients required only nasal cannula supplemental oxygen or no oxygen during admission. In addition, the substantial number of patients treated with remdesivir might have contributed to the lower death rate. Preliminary data from recent clinical trials suggest remdesivir use may be associated with reduced mortality (*18*,*19*).

The presenting symptoms and laboratory findings of the patients in our study are similar to those noted in the studies from China published earlier in the COVID-19 pandemic despite the finding that our patient population probably had greater racial and ethnic diversity (*6**–**9*). As seen in prior studies, respiratory complaints were the most common presenting symptoms; however, 5 (6.9%) patients in our study did not have respiratory symptoms of cough or shortness of breath. This finding emphasizes the importance of capturing nonrespiratory symptoms on COVID-19 screening questionnaires.

California in general, and the San Francisco Bay area in particular, have had a longstanding housing affordability crisis (*20*). Seven (9.7%) patients in our study lacked a safe place to isolate at home because of crowded living conditions (no separate bedroom), living with an immunocompromised person, or both. These circumstances also might have contributed to lower hospitalization death rates because patients with mild disease required hospital admission.

One important context for this study is the difference in the standard of care at the 2 institutions. When our institutions began seeing COVID-19 patients, the Centers for Disease Control and Prevention and the World Health Organization had published clinical care guidelines emphasizing supportive care as the standard of care and recommended using experimental therapies only as part of a randomized controlled trial (*21*,*22*). Both of our study sites are involved in clinical trials of the novel antiviral agent remdesivir but began enrolling at different times: March 14 at SUH and April 9 at ValleyCare. This difference in implementation led to nonuniform use of hydroxychloroquine, azithromycin, and zinc between SUH and ValleyCare. At both study sites, azithromycin was commonly prescribed to outpatients before admission, despite lack of evidence of clinical efficacy. Detailed national guidelines from the Society for Critical Care Medicine were published on March 20 and from the Infectious Disease Society of America on April 11 (*23*,*24*). Notable differences in the standard of care were seen even between our 2 affiliated hospitals, perhaps reflecting the initial lack of national guidelines.

Our study has several limitations. Overall case numbers were low, probably because of early and decisive public health interventions in our community (*25*). This observational study is not powered or designed to analyze treatment efficacy of the experimental therapies given. Analysis of the efficacy of remdesivir, received by most critically ill patients in this cohort, will be conducted as part of a multisite clinical trial. Four patients remained hospitalized at the conclusion of the study period, and final outcomes could therefore not be reported. Thus, the duration of hospitalization is weighted toward patients who had shorter admissions and outcomes of a subset of prolonged hospital courses were not captured. Our results most likely are not generalizable to hospitals with excessive COVID-19 caseloads or with fewer resources for high acuity patients.

In summary, we found that under nonsurge conditions, the overall death rate and the death rate for ICU patients were lower than those previously reported in the United States. The differences in treatment strategy between the 2 hospitals in this study highlight the need for standardized, well-publicized guidelines for new pathogens early on in an epidemic.
